# Shift in secretory profile of a pNET: from indolent glucagonoma to aggressive insulinoma – a case report

**DOI:** 10.3389/fendo.2025.1729451

**Published:** 2026-01-05

**Authors:** Sara Gil dos Santos, Raquel Calheiros, Joana Oliveira, Ana Paula Santos, Pedro Souteiro

**Affiliations:** 1 Department of Endocrinology, Portuguese Oncology Institute of Porto (IPO Porto), Porto, Portugal; 2 Porto Comprehensive Cancer Center (P.CCC), Porto, Portugal

**Keywords:** case report, pancreatic neuroendocrine tumor, insulinoma, glucagonoma, hypoglycemia

## Abstract

Pancreatic neuroendocrine tumors (pNETs) can change their hormonal profile over time, leading to new clinical syndromes that significantly impact prognosis and management. We report the case of a patient with a metastatic glucagon-secreting pNET who, after 14 years of disease and multiple treatment lines, developed insulin hypersecretion and severe, treatment-refractory hypoglycemia. Despite several strategies, including diazoxide, somatostatin analogues, glucocorticoids, everolimus, peptide receptor radionuclide therapy, and continuous glucose and glucagon infusions, glycemic control was not achieved, and the patient ultimately died from treatment complications. This case highlights the clinical challenges of managing metachronous hormonal syndromes and the importance of long-term endocrine follow-up in pNET patients. It also emphasizes the limitations of current therapeutic strategies and the urgent need for new treatment options, including alpha-emitting radiopharmaceuticals, which may offer improved disease and symptom control in advanced, insulin-secreting pNETs.

## Introduction

Pancreatic neuroendocrine tumors (pNETs) originate from pancreatic islet neuroendocrine cells and are functioning in about 10-40% of cases, with excess hormonal production associated with a clinical syndrome (1, 2). In functioning pNETs, morbidity and mortality are attributed not only to the local invasion and distant spreading, but also to the specific clinical manifestations of hormone excess (3–5).

Insulinomas, located in the pancreas in more than 99% of cases, are the most common functioning pNET (6). They account for 35-40% of cases and present with clinical manifestations related to the excess insulin production (3). Glucagonomas are rare, representing 1-2% of all pNETS and most patients present with typical skin lesions (necrolytic migratory erythema), new onset or worsening diabetes and weight loss (7).

Although rare, pNETs can secrete several hormones simultaneously or develop new functioning syndromes over time. Metachronous hormonal syndromes (MHs), defined as hormonal secretion not present at the time of initial pNET diagnosis, occur in 3-6% of cases and are associated with tumor progression and poorer prognosis (1, 8).

The majority of insulinomas are indolent, small, and localized, with cure rates after surgical ressection of 70-100% and 5-year survival rates approaching 94–100% (9, 10). On the other hand, 4-14% of insulinomas are aggressive, presenting with metastatic disease, with 5-year survival rates of around 24-67% (9, 11–13). Despite several treatment options for hormonal hypersecretion or structural disease control, that include diazoxide, somatostatin analogs (SSA), hepatic arterial embolization, targeted therapies, peptide receptor radionuclide therapy (PRRT), and chemotherapy, patients with aggressive insulinomas still present with unfavorable outcomes (11).

We present a case of a patient with a stage IV glucagon-producing pNET that, after 14 years and different treatment modalities, presented with new onset insulin secretion and refractory hypoglycemia. Our aim is to make physicians aware of the possible changes in pNET secretion pattern, to highlight the difficulty of managing refractory hypoglycemia in patients with metastatic insulinomas and to discuss unmet needs and future prospectives in treating these patients.

## Case presentation

We present a 69-year-old patient, referred to our Diabetes Clinic for hypoglycemia. The patient reported diabetes mellitus of 10 years’ duration, managed with insulin therapy until 3 months prior. At that point, insulin had been progressively tapered and eventually discontinued due to recurrent episodes of symptomatic hypoglycemia, which persisted despite complete insulin therapy withdrawal. He reported several capillary blood glucose measurements around 30 mg/dL and significant neuroglycopenic symptoms. This led him to adhere to a strict feeding schedule, consuming food every two hours, including simple sugars, to correct hypoglycemia and prevent loss of consciousness.

His diabetes was interpreted in the context of a G2 metastatic glucagon-secreting pNET, [glucagon at diagnosis of 2535 (100–190) pg/mL], with low volume hepatic and lymph node involvement on computed tomography (CT) imaging, diagnosed 14 years prior. Since the initial diagnosis, the patient had undergone multiple lines of treatment due to disease progression, including gemcitabine-based chemotherapy (from 2009 to 2010), long-acting first-generation somatostatin analogues (lanreotide, from 2011 to 2018), five cycles of peptide receptor radionuclide therapy (PRRT) with [^177^Lu]Lu-DOTA-TATE (3 cycles in 2011, and 2 additional cycles, in 2016), and targeted therapy with everolimus (from 2018 to 2020). At his first appointment at our Diabetes Clinic, in 2023, the patient was on Sunitinib, at a dose of 37.5mg/daily and reported no other chronic medication. There was no additional relevant personal or family medical history, and physical examination was unremarkable.

Considering the severe hypoglycemia episodes even after insulin discontinuation, the hypothesis of new-onset hyperinsulinemia was considered and bloodwork was performed, confirming inappropriate hormonal production (**Table 1**). The patient was given 25 mg of diazoxide 4 times daily. Interstitial continuous glucose monitoring revealed a time below range (TBR) of 35% after 1 week, with a considerable amount of hypoglycemia episodes confirmed through capillary blood glucose.

**Table 1:** Initial bloodwork.

Laboratory test	Value	Reference range
Glucose	36	76–115 mg/dL
Creatinine	0.71	0.67-1.17 mg/dL
Albumin	38	38–53 g/L
Total proteins	62	64–83 g/L
Aspartate aminotransferase	106	<39 U/L
Alanine aminotransferase	49	<42 U/L
Alkaline phosphatase	90	42–128 U/L
Gamma-glutamyl transferase	197	7–52 U/L
Sodium	138	135–145 mmol/L
Potassium	4.0	3.8-5.0 mmol/L
Insulin	39.2	2.6-24.9 uUI/mL
C peptide	6.95	1.1-5.0 ng/mL

Despite diazoxide dosage up titration, the patient continued to experience recurrent symptomatic hypoglycemia. In an attempt to suppress insulin secretion, long-acting somatostatin analogues were reintroduced: initially lanreotide 120 mg every 28 days, later replaced by pasireotide 60 mg every 28 days, due to its more pronounced hyperglycemic effect. However, none of these measures were particularly useful.

**Figure 1:**
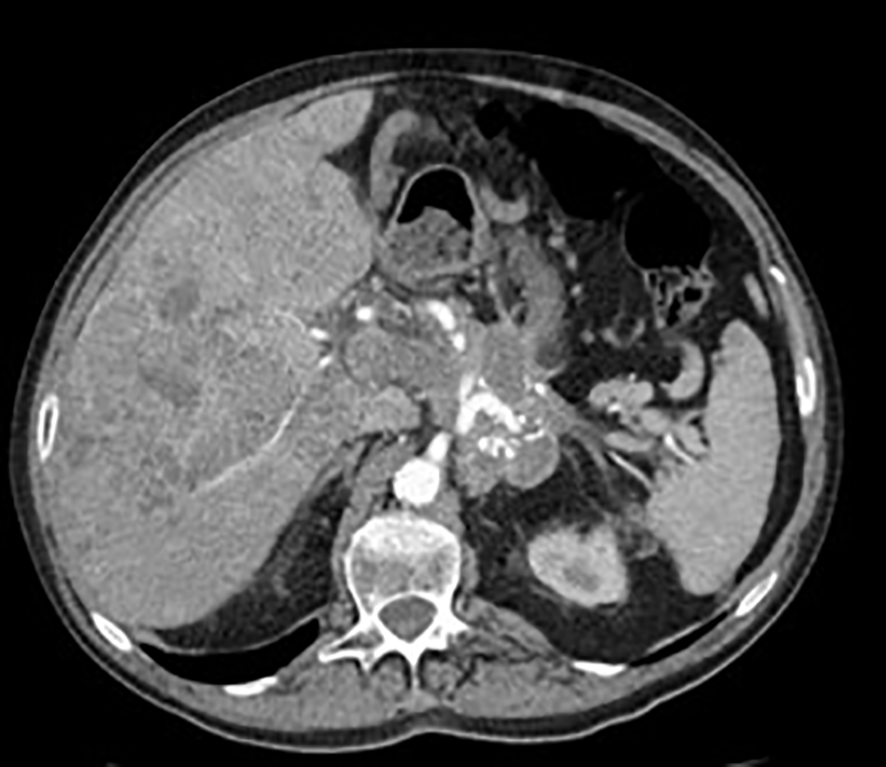
Abdominal CT scan performed at the time of insulinoma diagnosis showing extensive liver metastases.

**Figure 2:**
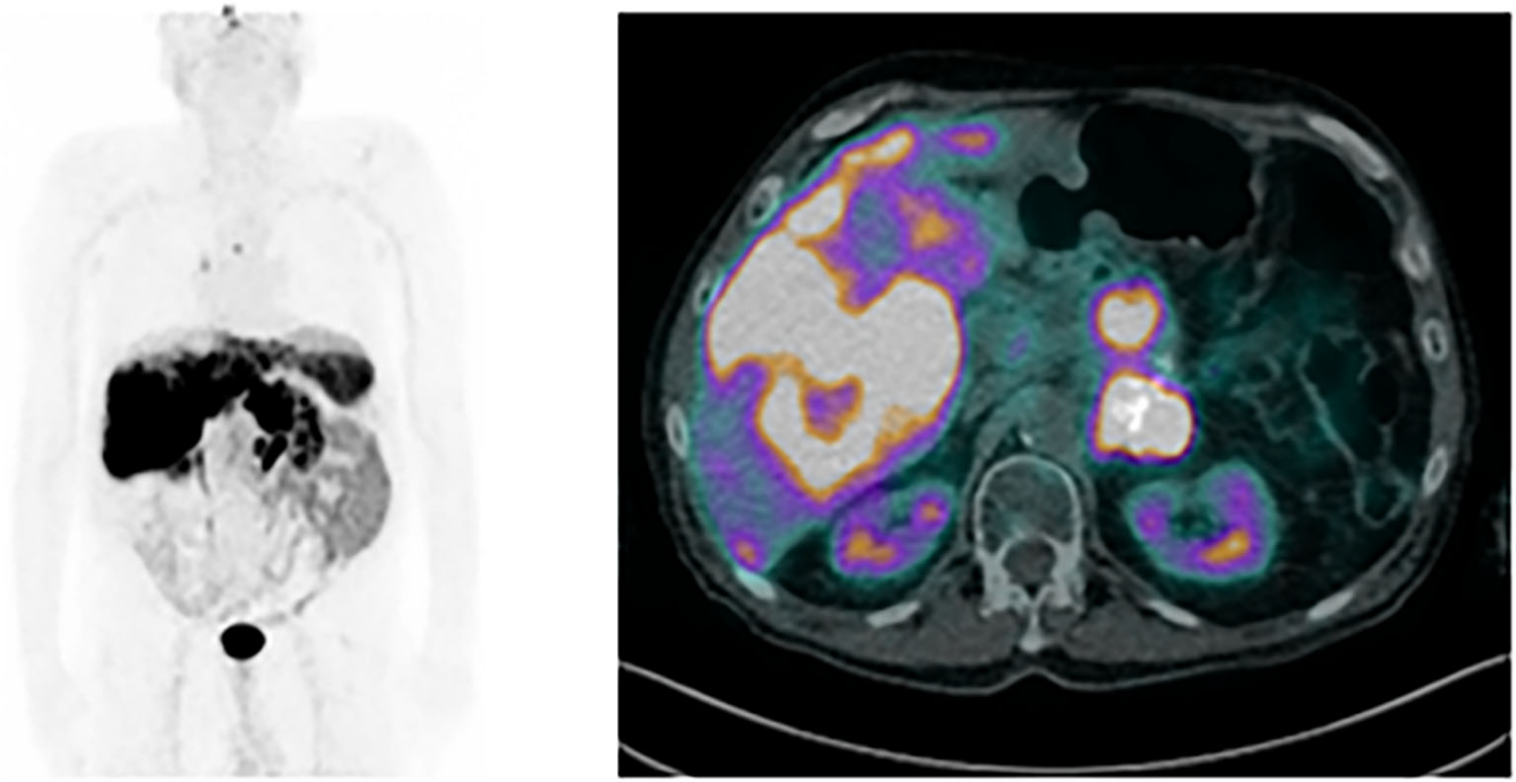
[^68^Ga]Ga-DOTA-NOC PET/CT scan performed at the time of insulinoma diagnosis; on the left, the maximum intensity projection shows uptake in several hepatic lesions and discrete uptake in lymph node lesions, including the left thoracic para-aortic, interaortocaval, and left lumboaortic chains, and right perihilar region, compatible with metastatic disease; on the right, the axial fused PET/CT image shows heterogeneous uptake in several hepatic lesions, compatible with metastases.

To assess structural disease, an abdominal CT was performed (**Figure 1**), which showed hepatic disease progression, with an increase in number and size of metastases. A [^68^Ga]Ga-DOTA-NOC PET/CT scan showed an increased number of somatostatin receptor positive lesions in the liver, and stable pancreatic and regional lymph nodes involvement (**Figure 2**). Considering disease structural progression and the difficulties in controlling the insulin production by the tumor, sunitinib was stopped and other therapeutic options were pursued.

Given the favorable response to previous PRRT, and the increased somatostatin receptor expression in all lesions, 3 months after the insulinoma diagnosis, the patient underwent two additional cycles of [^177^Lu]Lu-DOTA-TATE (total cumulative activity of 43.29 GBq). Despite an initial glycemic profile improvement, with TBR of 7% after 1 month, hypoglycemia quickly worsened. Corticosteroids (prednisolone 40 mg daily) were then initiated to try to induce secondary hyperglycemia, but the patients’ clinical condition kept deteriorating further, with several episodes of severe hypoglycemia. Admissions to the emergency department (ER) became increasingly frequent, with the patient sometimes visiting twice on the same day due to rapid recurrence of hypoglycemia following initial correction with intravenous glucose and dextrose infusion.

Approximately five months after the diagnosis of insulinoma, given the high frequency and severity of hypoglycemic episodes, outpatient care was considered no longer feasible or safe, and the patient was admitted for inpatient management.

Initial management included IV dextrose infusion, short-acting somatostatin analog therapy (subcutaneous octreotide 50mcg 3 times daily) and continuous intravenous glucagon infusion (0.25 mg/h), along with nutritional support optimization. Corticosteroid therapy was intensified (with a maximum dose of dexamethasone 12mg/daily) and subsequently down titrated to balance glycemic control and the appearance of side effects (weight gain, cushingoid miopathy, peripheral edema and easy bruising). Everolimus was reintroduced mainly for its hyperglycemic properties, with limited clinical benefits.

Considering the wide range of systemic therapies that were implemented without clinical benefit, liver directed therapies were considered. After discussion with the Nuclear Medicine Department, hepatic radioembolization was contraindicated due to the presence of a considerable hepatic arteriovenous shunt that precluded its safe administration.

The patient remained hospitalized for over two months, due to the recurrence of symptomatic hypoglycemia despite repeated administration of 30% intravenous glucose. Brief 30-minute interruptions in the glucose infusion were attempted to test for a possible hospital discharge, but hypoglycemia (~40mg/dL) with loss of consciousness quickly ensued.

**Figure 3:**
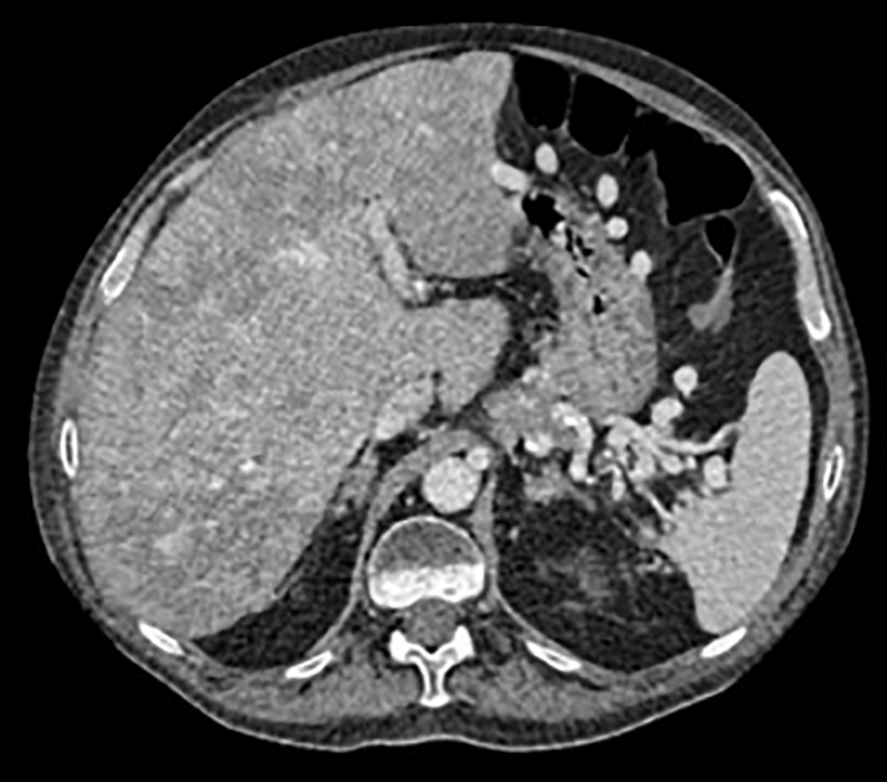
Abdominal CT scan performed 6 months after the insulinoma diagnosis showing progression of hepatic disease, with innumerable confluent focal lesions involving the entire parenchyma.

**Figure 4:**
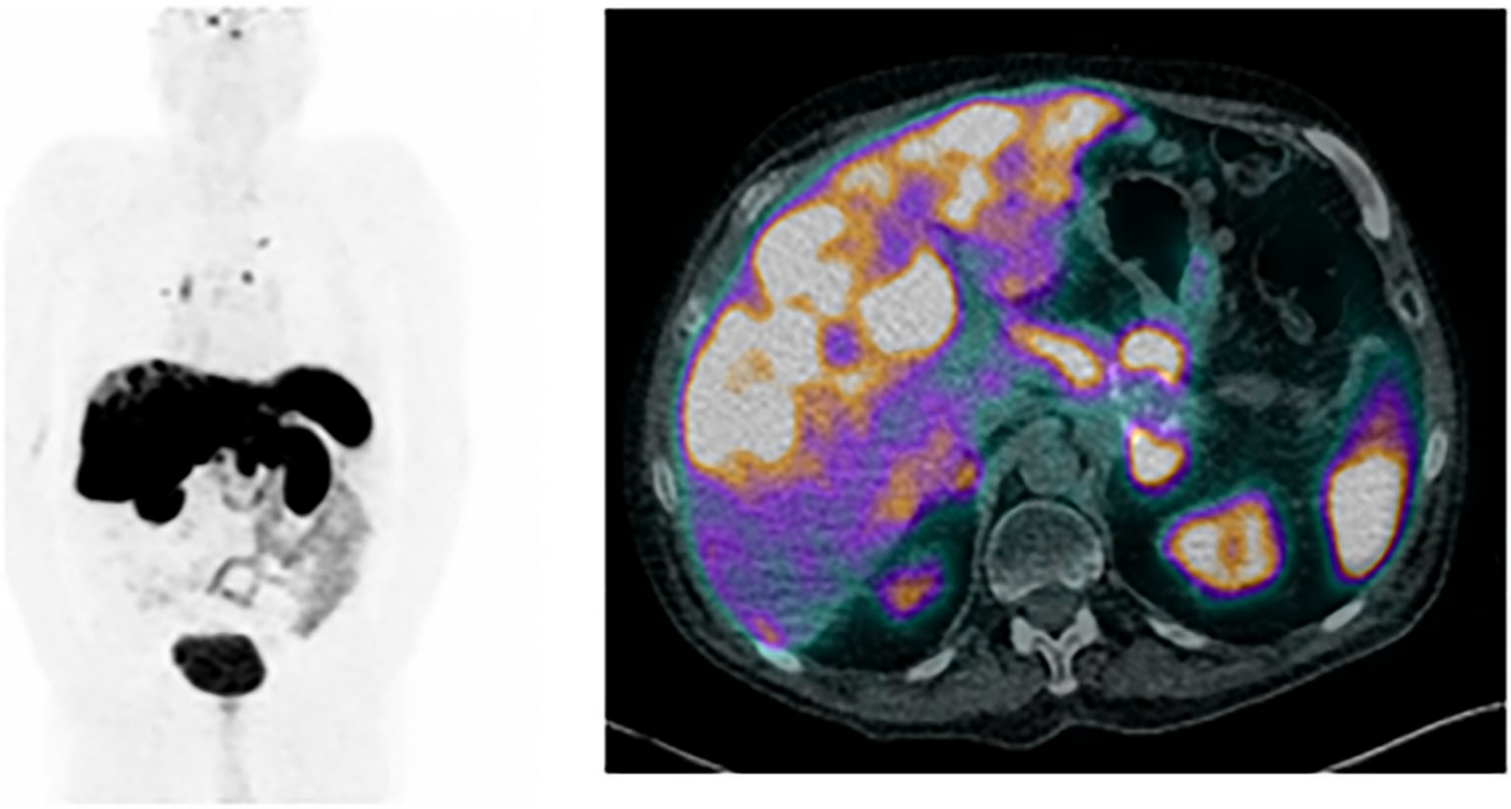
[^68^Ga]Ga-DOTA-NOC PET/CT scan performed 6 months after insulinoma; on the left, the maximum intensity projection shows uptake in pulmonary, hepatic and lymphatic lesions (including in the left internal mammary chain, right lower paratracheal and right hilar nodal stations, and lumboaortic chains), compatible with metastases, with an increase in hepatic disease burden, compared with the previous exam; on the right, axial fused PET/CT images shows an increase the number and size of several hepatic lesions, compatible with metastases.

An abdominal CT scan (**Figure 3**) and a [^68^Ga]Ga-DOTA-NOC PET/CT scan (**Figure 4**), performed approximately 6 months after insulin autonomous production was first detected, showed further disease progression, with new liver, pulmonary and lymph node metastasis. The patients’ condition worsened following a nosocomial pulmonary infection, and he passed away.

## Discussion

We present a patient with a glucagon-secreting pNET with metachronous insulin hypersecretion in the context of disease progression, which lead to treatment-refractory hypoglycemia and death. Several treatment strategies were used to no avail and the patient ended up passing away 6 months after the change in hormonal secretion pattern was detected.

The mechanisms through which changes in hormonal secretion occur in pNETs remain incompletely understood (1, 5). Pancreatic cell plasticity and the capacity for reprogramming may allow shifts in hormone production under certain stimuli (14–16). Moreover, the coexistence of different tumor clones, with different secretory potential, could contribute to this phenomenon (5). Targeted therapies such as sunitinib have also been implicated in inducing phenotypic changes in pancreatic neuroendocrine tumor cells, through epigenetic modulation (5).

Nevertheless, an abrupt change in clinical behavior in patients with neuroendocrine pancreatic tumors who develop MHS is strongly associated with underlying tumor dedifferentiation and increased aggressiveness. This phenomenon typically arises in the context of disease progression and is frequently accompanied by a significant increase in the Ki-67 proliferation index, reflecting a shift toward a higher tumor grade and more aggressive biology, with worse overall survival (5, 8, 17–20).

Crona et al. (5) showed, in a retrospective study with 323 patients with pNETs, that 4,3% presented MHs throughout the course of the disease. All patients showed progression within 3 months of hormonal syndrome detection. De Mestier L. et al. (17) reported, in a retrospective multicenter study with 435 patients with pNET, that 3,4% had MHs, linked to progressive disease and increase in Ki-67% indexes. In both studies, the patients had metastatic disease prior to the diagnosis of MHs, and insulin secretion related to worse prognosis, compared to other hormonal syndromes. Other retrospective studies have shown mixed results in terms of prognosis, when comparing different functioning NETs (21, 22).

Although cases of metachronous insulin secretion have been described in previously nonfunctioning and functioning pNETS, as far as we know, only 2 previous cases in literature have reported insulin secretion after a glucagonoma diagnosis (5, 23), a curious switch considering the opposite biological functions of the two hormones.

Despite the availability of several treatment options, agents approved for refractory hypoglycemia have limited efficacy and tolerability (24). Initial management typically includes dietary measures (9) and pharmacologic options like diazoxide and glucocorticoids, but these can be limited by adverse events (9, 25–28). In selected cases, debulking surgery or locoregional liver-directed therapies can be considered, to reduce tumor burden and improve glycemic control (9).

First generation SSA are effective in hormone secretion suppression, as well as tumor growth control (9, 26). Pasireotide, a second generation SSA, although currently not approved for the treatment of pNET, has been proposed as an alternative in patients with refractory hypoglycemia (9). While pasireotide has not demonstrated a definitive antiproliferative effect (29), its hyperglycemic effect, mediated through its particular affinity for somatostatin receptors 5 (SSTR5), which results in suppression of insulin secretion from pancreatic cells (11), proves therapeutically advantageous in insulinoma (11, 26, 30). Several case reports have documented the clinical benefits of pasireotide in managing refractory hypoglycemia, including in patients with aggressive insulinoma (11, 24, 30).

Everolimus, a mammalian target of rapamycin (mTOR) inhibitor, has a proven antiproliferative effect in patients with metastasized insulinomas (9). Hyperglycemia is also a common side effect, resulting not only from impaired insulin secretion from normal pancreatic cells and increased insulin resistance (31), but also through the specific suppression of pathologic insulin secretion from tumor cells in insulinoma patients (32–35).

PRRT with [^177^Lu]Lu-DOTA-TATE, is also approved for gastroenteropancreatic NETs with SSRT expression. Multiple retrospective series and case reports have shown that PRRT can lead to sustained control of hypoglycemia in the majority of patients, with symptomatic improvement rates above 90% (9, 36, 37). A case series from our center reported imaging improvement in 3 out of 4 patients with inoperable metastatic insulinoma after PRRT, and clinical improvement in all of them (38).

In the case of our patient, support measures showed little efficacy in controlling hyperinsulinism. SSA were introduced, but even pasireotide showed little clinical benefit. PRRT showed a small and short-lasting improvement, and everolimus did not exert its typical hyperglycemic effects. Repeated episodes of severe hypoglycemia led to the patients’ admission for implementation of other measures such as IV dextrose, glucagon infusion, short acting subcutaneous SSA and high dose corticosteroids, which still proved insufficient.

The relentless tumor progression highlights the pressing need for novel and more effective therapeutic options in the management of advanced insulin-secreting pNETs.

PRRT with alpha-emitting radioisotopes like ^225^Ac-DOTATATE has shown promising experimental results in advanced NETs. Beta-emitting radioisotopes (such as ^177^Lu, which we used in our patient) emit particles with low energy and long tissue penetration range, useful in the treatment of larger tumor masses, but with a higher risk of collateral damage to the surrounding, healthy, tissue. They also lead to single-strand DNA damage, which is more easily reparable. On the other hand, alpha-emitting radioisotopes (like ^225^Ac), emit particles with high energy and a very short penetration range, which minimizes the damage caused to the surrounding tissues, and lead to double strand-DNA damage and irreversible cell death, making it useful for targeting smaller, or resistant disease, or beta-PRRT refractory tumors (39, 40). Ballal et al. (41) showed improved survival outcomes and quality of life, with ^225^Ac-DOTATATE targeted alpha therapy, in 32 patients with metastatic gastroenteropancreatic NETs who had exhausted or were refractory to [^177^Lu]Lu-DOTA-TATE. Partial response was observed in 62.5% of patients with no disease progression or death during 8 (3-13) months of follow-up. In our patient, despite tumor progression, [^68^Ga]Ga-DOTA-NOC PET/CT still showed strong tumoral radiopharmaceutical uptake, making it potentially responsive to [^225^Ac]Ac-DOTA-TATE. Unfortunately, this is still only available as an experimental treatment modality in the context of clinical trials, and not available in our country.

PRRT using SSTR receptor antagonists has also shown promising results in metastatic disease. SSTR agonists, such as DOTA-TATE, bind to SSTR, and activate them, resulting in inhibition of hormone secretion and antiproliferative effects (42). SSTR antagonists, such as DOTA-LM3, bind to SSTRs and block receptor activation (43). Despite showing no internalization into tumor cells, unlike SSTR agonists, SSTR antagonists have demonstrated a higher tumor uptake and longer tumor retention time, likely due to a larger number of recognizable binding sites (44, 45). Baum et al. (45) described a cohort of 51 patients with progressive, metastatic neuroendocrine neoplasias, most of them with no or low uptake in [^68^Ga]Ga-DOTA-TOC or DOTA-TATE PET/CT, but with tumor uptake in [^68^Ga]Ga-NODAGA-LM3, treated with [^177^Lu]Lu-DOTA-LM3. This led to a disease control rate of 85.1%, with partial response in 36.2% and stable disease in 48.9%. Complete remission was achieved in 4.3% of patients, and partial remission in 44.7%. The treatment was well tolerated and effective even in patients with low or absent SSTR agonist binding, suggesting utility in cases refractory to conventional SSTR agonist-based PRRT, but it remains investigational and is not yet included in major clinical guidelines or approved for routine clinical use.

Additionally, therapeutic approaches to hyperinsulinemic hypoglycemia in clinical development include the human monoclonal antibody, RZ358 (ersodetug), which acts as a negative allosteric modulator of the insulin receptor, reducing insulin binding, and has been shown to reverse refractory hypoglycemia in a patient with malignant insulinoma (46). Alpelisib, an α-selective phosphatidylinositol 3-kinase (PI3K) inhibitor, plays a crucial role in insulin signaling, and has been used in patients with refractory congenital hyperinsulinism and non-islet cell tumor hypoglycemia (47, 48). The GLP-1 receptor antagonist exendin-9-39 (avexitide), which blocks the effect of GLP-1, decreasing insulin secretion and mitigating hypoglycemia, has shown promising results in patients with post bariatric hypoglycemia (49).

This case underscores the critical role of endocrinology in the long-term follow-up of pNETs. In our patient, the decline in quality of life and eventual death, primarily resulted from uncontrolled hypoglycemia due to hormonal hypersecretion, rather than from the tumor progression in itself. Despite the available therapeutic options for this disease, significant unmet needs remain, and new drugs and radiopharmaceuticals currently in development are eagerly anticipated. Careful clinical surveillance in pNETs is crucial, given the potential for evolving hormonal secretion profiles, which can significantly impact morbidity and mortality in these patients.

## Data Availability

The original contributions presented in the study are included in the article/supplementary material. Further inquiries can be directed to the corresponding author.
